# MicroRNAs as Potential Biomarkers and Therapeutic Targets in Ischemic Stroke from the Perspective of Inflammation

**DOI:** 10.2174/011570159X380644250707075513

**Published:** 2025-07-30

**Authors:** Nai-He Chen, Jia-Xin Ren, Guang-Jian Li, Xin Sun

**Affiliations:** 1 Department of Neurology, The First Hospital of Jilin University, Changchun, 130021, China

**Keywords:** Ischemic stroke, microRNAs, immune cells, inflammation, peripheral immune cells, microglia

## Abstract

Ischemic stroke, triggered by the interruption of cerebral blood flow, initiates a complex inflammatory process involving both brain-resident and peripheral immune cells. Microglia, the primary brain-resident immune cells of high heterogeneity, regulate central nervous system inflammation upon activation. Activated microglia are commonly classified into two predominant phenotypes (pro-inflammatory M1 and anti-inflammatory M2), which exert dual effects through the secretion of distinct cytokine profiles. Peripheral immune cells, including monocytes, macrophages, and neutrophils, contribute to stroke pathogenesis and progression *via* diverse inflammatory mechanisms. Multiple microRNAs regulate the inflammatory dynamics of ischemic stroke across all phases by modulating both brain-resident and peripheral immune cells. MicroRNAs play a pivotal role in the activation and polarization of microglia, as well as cytokine release. Furthermore, microRNAs modulate the activation and extravasation processes of peripheral leukocytes by enhancing or attenuating signaling pathways. These mechanisms suggest that microRNA alterations could be biomarkers for predicting, diagnosing, and prognosticating ischemic stroke. Additionally, microRNA modulation offers potential as a therapeutic strategy for the treatment of ischemic stroke.

## 1 INTRODUCTION

Stroke is the second leading cause of death globally, imposing a significant burden on patients and society [1]. Resulting from damage to cerebral blood vessels, stroke is characterized by neurological dysfunction lasting more than 24 hours, supported by neuroimaging evidence. Ischemic Stroke (IS), responsible for about 87% of all strokes [2], begins with the obstruction of blood vessels supplying the brain. According to the Trial of Org 10172 in Acute Stroke Treatment (TOAST) classification, IS can be categorized into five subtypes: Cardioembolic Stroke (CARD), Large-Artery Atherosclerotic stroke (LAA), Lacunar stroke (LAC), Stroke of Other Determined Etiology (SOE), and Stroke of Undetermined Etiology (SUE).

Currently, the accuracy and availability of predictive, diagnostic, and prognostic modalities for IS remain suboptimal, adversely impacting clinical outcomes. The Framingham Stroke Risk Score (FSRS), the most commonly used predictive tool, often overestimates the risk of cerebrovascular events [3, 4]. In current clinical practice, the diagnosis of IS primarily relies on clinical assessments supplemented by neuroimaging [5]. Computed Tomography (CT) is the recommended initial neuroimaging procedure for diagnosing IS [6], yet it produces false negatives in approximately half of the cases [7]. Diffusion-Weighted Imaging (DWI), a highly sensitive diagnostic tool for stroke, is constrained by lengthy procedures, high costs, and limited availability in remote areas [8]. Consequently, 2-26% of medical interventions for patients are delayed. Such delays contribute to poor clinical outcomes, elevated stroke recurrence risk, and high mortality rates [9, 10]. Hence, novel diagnostic biomarkers are crucial in addressing these critical limitations.

Reperfusion therapy, the first-line treatment for salvaging the penumbra during the acute phase of stroke, primarily involves recombinant tissue Plasminogen Activator (rtPA) and mechanical thrombectomy [11, 12]. However, less than 30% of patients with stroke qualify for reperfusion therapy due to strict exclusion criteria and a narrow time window for intervention [13-15]. Furthermore, its use is constrained by the risks such as ischemia-reperfusion injury, hemorrhagic complications, and inadequate neuroprotection [16, 17]. Given the harmful impact of IS, accurate biomarkers and refined therapeutic strategies are urgently needed.

IS is initiated by a sudden deprivation of cerebral blood flow, resulting in a subsequent oxygen and glucose shortage. The inflammatory process of IS engages multiple groups of immune cells [18], including both innate and adaptive immunity, in the brain and systemically. Most of these cells are multifunctional and regulate each other in different phases. Thus, monitoring and targeting the immune system is also an attractive strategy for diagnosing and treating IS, with lower risks of complications and better neuroprotective effects [19]. Emerging research has identified that multiple microRNAs (miRNAs) are involved in the inflammatory process of IS, exhibiting high stability in plasma and providing opportunities in diagnosing and treating cerebral infarction [20-23].

miRNAs are short, single-stranded, non-coding RNAs, approximately 22 nucleotides long, that regulate gene expression at the post-transcriptional level [24]. They regulate the expression of target genes by complementary binding to messenger RNAs (mRNAs), which leads to degradation of mRNAs and inhibition of translation, resulting in altered levels of target proteins [5, 25].

miRNAs are crucial in regulating immune cells in the brain and peripheral circulation during the IS process. These miRNAs can either stimulate or suppress inflammatory events. Altered miRNA expression profiles during IS, coupled with their stability in circulation, position them as promising biomarkers of IS [22, 26]. Additionally, miRNA-based interventions are emerging as potential therapeutic targets for IS treatment [27, 28]. This review explores the role of miRNAs in IS-associated inflammatory events, emphasizing their interactions with various immune cell types, and analyzes the prospects of miRNAs as biomarkers and therapeutic targets for IS.

## 2 METHODS

This review focuses on multiple miRNAs that mechanistically regulate brain-resident and peripheral immune cells during ischemic stroke, as well as their potential as biomarkers and therapeutic targets. To achieve this, a comprehensive literature search was conducted across PubMed, Scopus, and Web of Science databases, employing MeSH-indexed terms: (“microRNAs” OR “miRNA”) AND (“ischemic stroke” OR “cerebral infarction”) AND (“immune cells” OR “neuroinflammation”). Inclusion criteria prioritized peer-reviewed original research articles published within the last decade (2014-2024), excluding non-English publications.

## 3 PATHOGENESIS OF INFLAMMATION IN ISCHEMIC STROKE

**Fig. (1):**
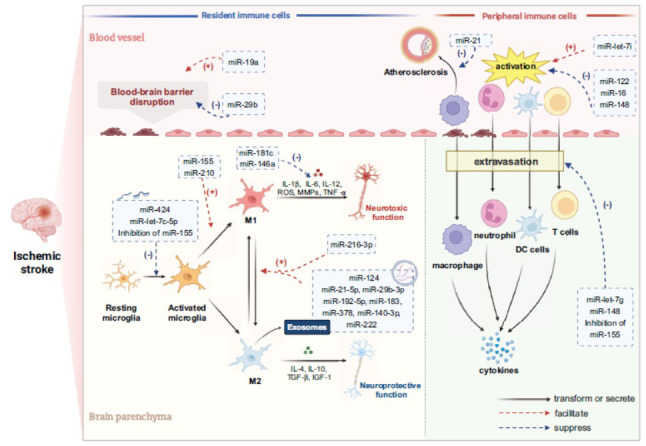
Neuroinflammation and the regulation of miRNAs in cerebral ischemic injury. After ischemia, circulating leukocytes are activated and extravasate into the brain parenchyma due to blood-brain barrier disruption. Meanwhile, damaged brain cells release DAMPs, activating resident immune cells such as microglia. Activated microglia polarize into two phenotypes, pro-inflammatory M1 and anti-inflammatory M2. These resident and peripheral immune cells exert their effects by secreting regulatory cytokines, ROS, or MMPs, or directly phagocytosing debris. miRNAs regulate every step of the immune response (red dash lines indicate facilitation; blue dash lines indicate suppression). miR-424, let-7c-5p, and the inhibition of miR-155 suppress microglial activation. miR-210 promotes polarization towards M1. miR-146a and miR-181c suppress the secretion of cytokines from M1. M2 secrete exosomes containing miRNAs, including miR-124, miR-21-5p, miR-29b-3p, miR-192-5p, miR-183, miR-378, miR-140-3p, and miR-222, which facilitate polarization from M1 to M2. Some miRNAs, such as miR-19a, promote blood-brain barrier disruption, while others, such as miR-29b, suppress it. From the perspective of peripheral immune cells, miR-let-7i promotes leukocyte activation, while miR-122, miR-16, and miR-148 suppress this process. miR-148, miR-let-7g, and the inhibition of miR-155 alleviate peripheral leukocyte infiltration. Furthermore, a low level of miR-21 in macrophages promotes macrophage apoptosis and exacerbates inflammation. (**Abbreviations**: miRNAs: microRNAs; DC: Dendritic cells; IL-1β: interleukin-1β; IL-4: interleukin-4; IL-6: interleukin-6; IL-12: interleukin-12; IL-17A: interleukin-17A; IL-23: interleukin-23; TNF-α: tumor necrosis factor-α; MMPs: matrix metalloproteinase; ROS: reactive oxygen species; TGF-β: transforming growth factor-β; IGF-1: insulin-like growth factor 1). Created in BioRender. Jin, J. (2025) https://BioRender.com/kg5y2sj.

Inflammation plays a critical role in all pathophysiological stages of IS, encompassing deprivation of blood supply, brain damage, subsequent repair [29], and ischemia-reperfusion injuries. The immune system primarily mediates inflammation. Innate immunity exerts both beneficial and detrimental effects during the acute phase, whereas adaptive immunity drives chronic inflammation and cytotoxicity [30]. During the acute phase, inflammatory mediators released by immune cells and necrotic tissue initially play a protective role by eliminating threats. However, persistent stimuli amplify inflammatory cascades, leading to secondary brain tissue damage (Fig. **1**). Simultaneously, harmful signals released into circulation exert an immunosuppressive effect on systemic immunity, resulting in post-stroke infections, which are a major cause of morbidity and mortality among patients with stroke [19].

This article primarily focuses on innate immunity, which predominates during the Acute IS phase (AIS). Innate immune cells involved in AIS are classified into brain-resident and peripheral immune cells. Microglia are the primary brain-resident immune cells, whereas peripheral immune cells consist of neutrophils, monocytes, macrophages, Dendritic Cells (DCs), and specific lymphocyte subsets (Fig. **1**). These immune cell groups interact through multiple inflammatory signaling pathways, jointly amplifying the stroke-induced inflammatory response [31].

### 3.1 Resident Immune Cells

#### 3.1.1 Microglia under Physiological Circumstances

Microglia, the primary brain-resident immune cells, are activated within hours of ischemic stroke and regulate central inflammation during progressive brain damage and subsequent repair [32, 33]. In light of the conflicting impacts of microglia in IS observed by diverse research, it has been demonstrated that microglia are a highly heterogeneous group of cells. Single-cell RNA sequencing (scRNA-seq) has identified a minimum of 13 distinct microglial states based on transcriptional profiles, 7 of which are associated with stroke [34]. It has been revealed that different microglial subsets have diverse properties, characterized by their unique clusters. Given the paucity of research on the interaction between miRNAs and distinct microglial subsets, a detailed discussion on microglia heterogeneity is beyond the scope of this review.

Under physiological conditions, microglia exhibit distinct functions during their developmental and adult stages. In their developmental stage, microglia play a phagocytic role, removing necrotic cells and remodeling neural tissue [35-37]. In their mature state, microglia remain quiescent, continuously scanning and monitoring their surroundings [38].

#### 3.1.2 Microglia in IS

During ischemia, microglia activate rapidly in response to signaling cues. They migrate to the infarcted area, altering their shape and function. After Middle Cerebral Artery Occlusion (MCAO), ramified microglia were observed within 6 hours and persisted for 7 days in the ischemic hemisphere [39]. In the infarcted area, microglia fragment within 12 hours of IS, significantly reducing their numbers after 24 hours [29]. Subsequently, microglia in the ischemic penumbra are activated by Lipopolysaccharide (LPS) or Interleukin-4 (IL-4) [40], with activation persisting for weeks after IS [41]. In later stages, microglia phagocytose dead cells and debris to promote stroke recovery [42]. However, they also phagocytose surviving ischemic neurons, exacerbating neuronal death around the infarct [43] (Fig. **1**).

Activated microglia promote neuroinflammation, which protects the brain from pathogens and neurotoxic agents while supporting tissue repair [44, 45]. Conversely, uncontrolled neuroinflammation exacerbates brain tissue damage through elevated glial activation, increased Blood-Brain Barrier (BBB) permeability, and peripheral immune cell infiltration [46, 47].

As previously mentioned, microglia exhibit contradictory roles in the pathological process of IS due to their dynamic and diverse states [34]. By focusing on their inflammatory activities, stroke-associated microglial subsets can be simplified into two phenotypes, M1 and M2. M1 microglia represents the pro-inflammatory phenotype, secreting pro-inflammatory cytokines (*i.e*., Tumor Necrosis Factor-α (TNF-α), IL-1β, and IL-6), Reactive Oxygen Species (ROS), and Matrix Metalloproteinases (MMPs) that promote inflammation, oxidative stress, and BBB disruption, thereby intensifying brain damage [48, 49]. Conversely, the anti-inflammatory M2 phenotype exerts protective effects by releasing anti-inflammatory cytokines such as IL-4, IL-10, Transforming Growth Factor-β (TGF-β), as well as the neurotrophic factor insulin-like growth Factor 1 (IGF-1), which reduce brain damage, promote functional recovery, and improve stroke prognosis [39, 50] (Fig. **1**).

Studies have shown that microglial polarization is closely linked to stroke progression. In the early stages of stroke, most recruited microglia adopt the M2 phenotype, exerting protective effects. As ischemia progresses beyond 1 week, microglia activation expands, and the M1 phenotype becomes dominant, aggravating brain damage and a worsening prognosis [39, 51]. Microglial polarization is regulated by multiple pathways, such as Nuclear Factor kappa B (NF-κB), Signal Transducer and Activator of Transcription (STAT) family members, Toll-Like Receptors (TLRs), Notch signaling pathways, and the thrombospondin A2R receptor [49].

Given the role of microglial M1/M2 polarization in inflammation, modulating polarization holds therapeutic potential for ischemic stroke, offering reduced complication risks and enhanced neuroprotection [52, 53]. Additionally, the M1-to-M2 phenotype ratio is a useful indicator of secondary brain injuries following cerebral ischemia [30, 54-57] (Fig. **1**).

### 3.2 Peripheral Immune Cells

#### 3.2.1 Monocytes and Macrophages

*Pathogenesis:* Intracranial Atherosclerosis (ICAS) contributes significantly to the pathogenesis of ischemic stroke. Monocytes and macrophages are the primary peripheral immune cells involved in the pathogenesis and progression of IS, engaging in various inflammatory processes (Fig. **1**). Macrophages alternate between two phenotypes resembling the polarization of microglia, which exert opposite inflammatory effects [58, 59].

Monocytes and macrophages contribute to the onset and progression of ICAS, a key pathological process in LAA, the primary IS subtype with the highest recurrence rate [60, 61]. The pathological process of ICAS primarily involves plaque formation, followed by rupture or growth, with monocytes and macrophages playing roles throughout all phases [25]. During this process, monocytes migrate into the subendothelial space and differentiate into macrophages, proliferating and engulfing oxidized Low-Density Lipoprotein (oxLDL). Within the lesion, predominant M1 macrophages are linked to the progression of ICAS, while M2 macrophages play a protective role [25]. Accumulated macrophages eventually transform into foam cells, a significant component of plaques that influence plaque stability [25]. During the progression of ICAS, local inflammatory infiltration of macrophages can destabilize atherosclerotic plaques, causing rupture, complete obstruction of blood flow, and subsequent brain tissue ischemia [25, 62]. If the plaque remains stable, it continues to grow due to the recruitment of monocytes to the lesion. Over time, the lumens narrow, leading to a chronic reduction in blood flow [25]. Additionally, the infiltration of monocytes into the microvasculature may contribute to the small-vessel LAC [63] (Fig. **1**).

*Progression and prognosis of IS:* Monocytes and macrophages infiltrate the ischemic brain through chemokine signaling (CCL2/CCR2) [64, 65]. In the early stages of ischemia, monocytes and macrophages exert a protective role by phagocytosing damaged brain cells. Later, monocyte and macrophage infiltration exacerbate brain damage, regulated by adhesion molecules, chemokines, and cytokines [66]. Monocytes and macrophages also play a crucial role in reducing the risk of hemorrhagic transformation after ischemic stroke [67].

#### 3.2.2 Neutrophils and Other Peripheral Immune Cells

Neutrophils play an essential role as peripheral immune cells in the pathophysiology of ischemic stroke, damaging the brain by producing reactive oxygen species, proteases, and cytokines, and activating the complement cascade (Fig. **1**). Recruitment and infiltration of neutrophils in and around vessels are triggered by sterile inflammation [18], leading to brain parenchymal damage and obstruction of blood flow.

During the early stages, neutrophils migrate and attach to endothelial cells by binding to adhesion molecules [67]. Subsequently, neutrophils extravasate from leptomeningeal vessels into the brain parenchyma across the BBB [18]. Meanwhile, damage to the BBB induces hemorrhagic transformation after ischemic stroke, further aggravating cerebral edema and neurological dysfunction [68]. Upon activation, neutrophils release proteases, reactive oxygen and nitrogen species, and inflammatory IL-1β and form neutrophil extracellular traps (NETs), which aggravate ischemic damage. Signs of neutrophil NETs formation inside and around the vessel lumen indicate that the Neurovascular Unit (NVU) is the primary site of neutrophil action in stroke [18]. The NVU facilitates communication between the peripheral immune system and the Central Nervous System (CNS) by regulating BBB permeability and subsequent leukocyte entry [69]. Furthermore, neutrophils can regulate and interact with other leukocytes, exhibit diverse phenotypes, and play important roles in several pathological conditions [70] (Fig. **1**).

Other peripheral immune cells involved in the onset and progression of IS include DCs, Natural Killer (NK) cells, and lymphocytes (Fig. **1**).

As antigen-presenting cells with high migratory capacity, DCs are found in the ischemic brain early after injury and persist for at least 7 days. Among DC subtypes, Xcr1-CD172+ cDC2s secrete IL-23, inducing IL-17 expression in γδ T cells and promoting neutrophil infiltration, contributing to ischemic brain injury in mice [71]. NK cells contribute to ischemic brain injury and are recruited by ischemic neuron-derived C-X3-C motif Chemokine Ligand 1 (CX3CL1). The local inflammatory effects of NK cells are mediated by their expression of IFN-γ and perforin [72].

In the context of innate immunity in IS, the detrimental function of T cells is associated with antigen-mediated activation. During the early stages of ischemia, Interferon-gamma (IFN-γ) produced by Cluster of Differentiation 4 (CD4)^+^ T cells induces macrophage TNF-α production. Simultaneously, IL-17A-secreting γδ T cells exacerbate ischemic injury by promoting neutrophil recruitment. Neutralizing IL-17A within 3 hours of stroke induction reduces infarct size and improves neurological outcomes [73]. TNF-α and IL-17A also enhance C-X-C motif Chemokine Ligand 1 (CXCL-1) secretion by astrocytes, a key neutrophil chemoattractant.

## 4 ROLES OF MIRNAS IN THE INFLAMMATION OF ISCHEMIC STROKE

As discussed above, inflammation in IS is mediated by the complex interplay of multiple cell groups within the CNS and periphery. As post-transcriptional regulators, miRNAs modulate both brain-resident immune cells (Table **1**) and peripheral immune cells (Table **2**), thereby influencing disease progression and recovery.

### 4.1 Role of miRNAs in Central Immune Cells

Research has shown that miRNAs are involved in various aspects of microglial function in IS, including the activation of resting microglia, polarization into different phenotypes, and exosome production (Table **1**).

#### 4.1.1 Activation of Resting Microglia

After cerebral ischemia/reperfusion, resting microglia are activated by LPS or IL-4 in the microenvironment, transforming into either the pro- or anti-inflammatory phenotype. Although microglial activation is an adaptive response to damage, its overactivation can exacerbate neuroinflammation and increase neuronal death by producing and distributing pro-inflammatory cytokines such as TNF-α, IL-1β, and IL-6 [69]. Recent studies have revealed that several miRNAs significantly regulate microglial activation and the microenvironment, reducing post-ischemic injuries. For instance, miR-424 suppresses microglial activation by targeting key activators of the G1/S phase transition and alleviates oxidative stress through the induction of nuclear factor erythroid 2-related factor 2 (Nrf2) expression [74, 75]. Similarly, the overexpression of let-7c-5p decreases microglial activation by directly targeting the caspase 3 pathway [76]. The inhibition of miR-155 reduces the early transient harmful effects of activated microglia after ischemic stroke by deactivating STAT-3 and suppressing the Janus Kinase-Signal Transducer and Activators of Transcription (JAK-STAT) signaling cascade [77, 78].

#### 4.1.2 Polarization of Activated Microglia

From the perspective of inflammation, activated microglia could be defined into two different phenotypes (M1 and M2). The function of each phenotype and microglia polarization can be targeted for modulation.

As the pro-inflammatory phenotype, M1 aggravates the detrimental effects of stroke by releasing inflammatory cytokines, which several miRNAs can modulate through the NF-κB pathway and other signaling pathways. During stroke, miR-210 has been shown to upregulate the expression of pro-inflammatory cytokines and chemokines through regulating Sirtuin1 (SIRT1)/ NF-κB pathway [80, 81]. However, the upregulation of miR-210 may facilitate angiogenesis after cerebral ischemia, which is beneficial for patients’ prognosis [99, 100]. Other studies have demonstrated that intravenous administration of miR-210-loaded exosomes to the ischemic brain enhances the expression of Vascular Endothelial Growth Factor (VEGF) and promotes angiogenesis [101]. It has been observed that miR-181c is downregulated in both Oxygen-Glucose Deprivation (OGD)-activated microglia and the brain tissue of MCAO rat models; notably, its administration is protective [56, 82]. miR-181c inhibits the expression of TLR4, thereby suppressing the NF-κB pathway and reducing the production of pro-inflammatory cytokines [56, 82]. Studies have shown that the expression of miR-146a is upregulated following LPS stimulation [102], which exerts a protective effect in stroke [103]. miR-146a downregulates the expression of M1-related genes by inhibiting Interleukin-1 Receptor-Associated Kinase (IRAK1) /NF-κB signaling pathway, thereby mitigating the inflammatory response [83]. The M2 phenotype is neuroprotective by promoting brain tissue regeneration and repair after ischemia through miRNA-mediated regulation. Following IL-4 stimulation, the transition from M0 to M2 leads to significant upregulation of miR-145 and miR-124 and downregulation of miR-711 [84].

Furthermore, several miRNAs exert protective effects by inhibiting M1 microglia polarization and promoting M2 polarization through different pathways involving exosomes. For instance, M2 microglia-derived exosomes containing miR-124 were confirmed to facilitate neuronal survival by targeting Ubiquitin-Specific Protease 14 (USP14) [87], while exosomes containing miR-26a secreted by M2 microglia promote angiogenesis [104]. miR-21-5p and miR-29b-3p in exosomes drive M2 polarization while inhibiting M1 polarization by suppressing inducible Nitric Oxide Synthase (iNOS) mRNA expression and downregulating pro-inflammatory mediators [47]. Exosomal miR-192-5p, miR-183, miR-378, miR-140-3p, and miR-222 have been demonstrated to promote M2 polarization of microglia by inhibiting TNF or/and IL-1β expression [88-91]. Intracranial injections of miR-124 after stroke have been shown to shift microglia polarization from M1 to M2 by inhibiting the transcription factor CCAAT/Enhancer Binding Protein-α (C/EBP-α) and its downstream target protein, Purine Rich Box-1 (PU.1) [86].

As previously mentioned, the dominant microglial phenotype in the early stage of IS is M2. However, after 1 week, the M1 phenotype becomes more prevalent [105, 106]. miR-124 may regulate the extension of the M2 phase and shorten the M1 phase, thereby contributing to the balance between M1 and M2 phenotypes [86]. Moreover, miR-216-3p shifts microglia polarization from M1 to M2 phenotype by inhibiting the TLR/ NF-κB signaling pathway [92]. Uniquely, miR-155 has been reported to promote M1 and M2 polarization in microglia by inhibiting TGF-β-Activated Kinase 1-Binding Protein 2 (TAB2) [79].

### 4.2 miRNAs in Peripheral Immune Cells

miRNAs regulate peripheral leukocytes by modulating activation and extravasation processes, enhancing or attenuating signaling pathways. Specifically, miRNAs can regulate macrophage behaviors in atherosclerosis [25], which plays a role in the etiology of IS (Table **2**).

#### 4.2.1 miRNAs' Regulation of Leukocyte Activation

miRNAs may regulate the activation of peripheral leukocytes in IS. Several cellular blood miRNAs have been shown to modulate immune activation and inflammatory responses by targeting genes involved in TLR and NF-κB signaling [107]. Let-7i has also been shown to be involved in leukocyte proliferation and activation by targeting High Mobility Group Box 1 (HMGB1), CD86, and Interleukin-8 (IL-8) [93]. miR-122 regulates the expression of Danger-Associated Molecular Patterns (DAMPs) involved in immune activation following a stroke [94]. miR-148 alters cytokine production (IL-6, TNF-α, IL-12, CD70), T-cell proliferation, major histocompatibility complex II (MHC II) expression, and DCs' antigen presentation [94, 95]. miR-16 inhibits macrophage activation by targeting programmed cell death 4 (PDCD4) and regulating pro-inflammatory Mitogen-Activated Protein Kinase (MAPK) and NF-κB signaling [25].

#### 4.2.2 miRNAs' Regulation of Leukocyte Extravasation

Leukocyte extravasation refers to the leukocyte attaching to the subendothelial region and invading the brain parenchyma across the BBB, an essential step in the inflammatory response during IS. Several cellular blood miRNAs have been found to regulate genes involved in leukocyte adhesion and BBB disruption in patients with IS [107]. For instance, miR-148 regulates the expression of the adhesion molecule Lymphocyte Function-associated Antigen 1 (LFA-1) and MMP-10, MMP-13, and MMP-15 in leukocytes [96]. Mechanistic studies reveal that miR-19a induces MMP-3 overexpression through TLR2-dependent transcriptional activation. This pathological cascade may contribute to BBB disruption and vascular remodeling post-stroke [94]. miR-let-7g attenuates the recruitment and migration of macrophages by regulating the intracellular Ca^2+^-activated Protein Kinase C/ oxidized Low-Density Lipoprotein/ lectin-like LDL receptor-1 (PKC/oxLDL/LOX-1) pathway [25]. In patients with stroke and rats with MCAO, leukocytic miR-29b attenuates the inflammatory response by augmenting BBB integrity through Complement component 1q (C1q) and TNF-6 [98]. The inhibition of miR-155 decreases the mRNA and protein expression of Monocyte Chemotactic Protein 5 (MCP-5) and IL-3 in distal Middle Cerebral Artery Occlusion (dMCAO). These pro-inflammatory cytokines activate the adhesion and migration of monocytes through the vascular endothelium [77].

#### 4.2.3 miRNAs' Regulation of Macrophage Behaviors in Atherosclerosis

Several miRNAs critically regulate macrophage-mediated inflammatory responses in atherosclerosis. For instance, miR-21 regulates multiple aspects of atherosclerosis, including vascular inflammation, foam cell formation, apoptosis, and plaque necrosis. Mechanistically, low miR-21 levels in macrophages lead to upregulation of Mitogen-Activated Protein Kinase Kinase 3 (MAP2K3) and subsequent activation of the p38/c-Jun N-terminal Kinase/Extracellular-signal-Regulated protein Kinase (p38/JNK/ERK) signaling cascade, thereby promoting macrophage apoptosis and exacerbating inflammation [25]. Notably, exosomal miR-30c-2-3p derived from foam cells exacerbates neuroinflammation by activating the TGF-β/SMAD2 pathway, which aggravates cerebral injury during IS [108]. These findings collectively highlight miRNAs as pivotal mediators in the vicious cycle of IS-associated inflammation, linking immune dysregulation to tissue damage.

## 5 MIRNAS AS BIOMARKERS IN AIS

### 5.1 Predictive Biomarkers

Primary prevention of IS can reduce the impact of the condition by identifying and managing stroke risk factors. FSRS is widely used to predict the 10-year probability of cerebrovascular events in asymptomatic individuals. However, it tends to overestimate these events in the general population due to the exclusion of endothelial function [3, 4]. Since several miRNAs have been identified as crucial modulators of microvascular endothelial function, they may address the FSRS gap and improve its risk discrimination accuracy [6, 109]. Moreover, miRNAs are linked to the pathogenesis of other risk factors, which have been proposed as potential predictive markers for stroke [110]. For instance, miR-126 targets Vascular Cell Adhesion Molecule-1 (VCAM1), which promotes leukocyte adherence to the endothelium during atherosclerosis. Decreased expression of miR-126 upregulates VCAM1, exacerbating atherosclerosis and contributing to the progression of IS. Hence, miR-126 shows promise as a predictor [111]. An extensive cohort study also observed a 3-miRNA combination model, consisting of miR-1268b, miR-4433b-3p, and miR-6803-5p, with an Area Under the Curve (AUC) of 0.88, to predict future stroke events in asymptomatic adults [6, 112].

### 5.2 Diagnostic Biomarkers

At the onset of IS, an accurate and sensitive early-stage diagnosis is crucial for implementing prompt and appropriate treatment strategies, which are highly time-sensitive. As mentioned above, the current methods of IS diagnosis—CT and DWI—suffer from high false-negative rates and limited accessibility, respectively. Therefore, developing a non-invasive, rapidly accessible, and low-cost method for diagnosing strokes with high sensitivity and specificity is essential [113].

#### 5.2.1 Distinguishing Ischemic Strokes from Healthy Controls and Stroke Mimics

Abnormally expressed miRNAs in the blood of patients with IS have attracted significant attention as potential diagnostic or prognostic biomarkers [114, 115] (Table **3**). These miRNAs exhibit remarkable stability and resistance to harsh conditions, and their circulatory levels are closely associated with patient outcomes. For example, miR-125a-5p, miR-125b-5p, and miR-143-3p levels were increased within 24 hours in patients with AIS compared to those with Transient Ischemic Attacks (TIAs) and Healthy Controls (HC) [116]. Thus, the combination of these three miRNAs could serve as a diagnostic model for AIS, with remarkable sensitivity (85.6%), specificity (76.3%), and an AUC value of 0.90 [116]. In contrast, serum miRNA-221-3p and miRNA-382-5p were downregulated in patients with AIS within 6 hours and could serve as independent diagnostic biomarkers, with AUC values of 0.810 and 0.748, respectively [117]. Additionally, serum miR-124 expression in AIS patients was reduced at 24, 48, and 72 hours after onset compared to healthy controls, with AUC values of 0.9527 (24 hours), 0.9487 (48 hours), and 0.8668 (72 hours), respectively. Serum miR-124 levels increased in patients with AIS at 72 hours [118]. Decreased plasma miR-30a, miR-126, and let-7b expression were also reported in patients with AIS at 24 hours, 1 week, 4 weeks, and 24 weeks compared to healthy controls, with high AUC values. Specifically, the plasma let-7b levels were decreased only in patients with LAA but increased in other ischemic stroke subtypes [119].

As mentioned above, several miRNAs are involved in the inflammatory processes of IS. A recent integrated meta-analysis identified miR-320b and miR-320d as key miRNAs involved in IS-related inflammation [120]. Furthermore, miR-let-7i, which regulates leukocyte proliferation and activation, was significantly reduced in patients with ischemic stroke, and its levels were negatively correlated with stroke severity [93].

#### 5.2.2 Discriminating Between Ischemic Stroke Subtypes

Determining the precise etiology of AIS among its five subtypes is essential, as treatment approaches vary based on the underlying cause. Circulating microRNAs are reported to exhibit distinct post-stroke expression patterns across different subtypes (Table **3**). Receiver Operating Characteristic (ROC) analysis demonstrated that upregulated miR-125b, miR-125a, let-7b, and let-7e expression levels in CARD could distinguish this subtype from others [121]. Previous studies have consistently reported high expression of the miR-17 family in patients with small-vessel disease, but not in those with LAA, CARD, or SUE [122]. Additionally, miR-7-2-3p and miR-1908 were associated with LAA and LAC, but not with other IS subtypes [121, 123].

### 5.3 Prognostic Biomarkers

A prognostic biomarker for stroke is an indicator used to evaluate short- or long-term outcomes, including treatment efficacy, post-stroke complications, and stroke recurrence. As previously discussed, miRNAs are highly stable in circulation and play regulatory roles in multiple events post-stroke, as demonstrated by numerous studies [124].

A study found that the downregulation of miR-124 in serum collected 72 hours post-stroke correlated with unfavorable outcomes 1 month later, as measured by the National Institutes of Health Stroke Scale (NIHSS) and the Glasgow Outcome Scale (GOS) [5, 118]. At 3 months post-stroke, upregulated miR-29b in circulating blood within 72 hours of symptom onset was associated with favorable patient outcomes [125], including reductions in brain infarct and edema volumes. However, higher expression levels of miR-125b-5p, miR-124-3p, miR-192-5p, miR-16, and miR-223 in plasma samples collected 24 hours post-thrombolysis were found to correlate with poor outcomes in patients with AIS [126, 127]. In another study, elevated plasma miR-124-3p levels in patients with AIS within 24 hours of symptom onset were negatively associated with survival within 3 months of hospital admission, whereas miR-16 was positively associated with survival [128].

Hemorrhagic Transformation (HT) is a common complication of ischemic stroke, particularly following thrombolytic therapy, and significantly increases mortality and morbidity rates [6]. miR-21-5p (AUC: 0.677), miR-206 (AUC: 0.687), and miR-3123 (AUC: 0.661) regulate the expression of the basement membrane-degrading enzyme MMP-9 and have been shown to predict the risk of HT following CARD [129]. Stroke recurrence, a critical event influencing IS outcomes, is associated with the levels of atherosclerosis-related miRNAs, such as miR-17 [130].

## 6 MIRNAS AS POTENTIAL THERAPEUTIC TARGETS IN AIS

The pivotal roles of miRNAs in the inflammatory processes of IS and their potential as predictive, diagnostic, and prognostic biomarkers have been discussed. Numerous studies indicate that manipulating miRNA levels through mimics, activators, or inhibitors could be a therapeutic approach following stroke [6] (Table **4**). Intracerebroventricular injection of the miR-let-7c-5p mimic in MCAO mice has reduced ischemic/reperfusion brain injury and neurological deficits by suppressing microglial activation through the miRNA-mediated caspase 3 pathway [76]. In MCAO rats, miR-29b activators reduced brain infarct volume and edema [98]. Administration of miR-195 activators within 6 hours of IS has reduced the inflammatory response and infarct size in MCAO rats. Additionally, inhibiting miR-155 (48 hours post-MCAO), miR-377 (10 minutes post-MCAO), and miR-200c (1 hour post-MCAO) *via* injected inhibitors significantly reduced infarct volume and neurological impairment in MCAO rodents [77, 78, 131, 132].

Microglial M1/M2 polarization, a critical process in IS, appears to be a promising therapeutic target that miRNAs can modulate. Compared to traditional recanalization therapies, targeting the balance between M1 and M2 polarization reduces complication risks and offers superior neuroprotective effects [52, 53]. Upregulation of miR-424 or its restoration by activators (administered 10 minutes before MCAO) in MCAO mice reduces cerebral infarction volume and brain edema by inhibiting microglial activation, oxidative stress, and apoptosis [75]. Additionally, intracerebral injection of miR-124 in a stroke mouse model 48 hours post-stroke promoted microglial polarization from M1 to M2, contributing to the restoration of brain function [86].

However, identifying delivery systems that can cross the BBB while ensuring minimal side effects and reliable *in vivo* stability remains challenging [133]. Exosomes containing miRNAs targeted toward microglial polarization offer a potential solution to these delivery challenges [134]. In patients with AIS and rat models, exosomes mitigate neuroinflammation-induced brain damage by releasing hsa-miR-124-3p and miR-30d-5p to promote M2 microglial polarization [135, 136].

## CONCLUSION

AIS continues to pose a major challenge to global health, with substantial morbidity and mortality rates. The current diagnostic paradigm for AIS relies on neuroimaging confirmation combined with thorough clinical evaluation. However, this approach faces limitations, including prohibitive costs, limited availability in remote areas, and potential diagnostic delays that often preclude patients from receiving timely reperfusion therapy. As the first-line treatment for AIS, reperfusion therapy also presents clinical challenges due to narrow treatment eligibility criteria and an elevated risk of HT. Emerging evidence has revealed that the critical involvement of neuroinflammation in AIS pathogenesis is mediated by diverse immune cell populations. Targeting these immune cells could serve as a diagnostic biomarker candidate and a neuroprotective therapeutic strategy.

It has been proved that miRNAs regulate the inflammatory processes of IS by modulating both brain-resident and peripheral immune cells [22]. In microglia, miRNAs regulate the activation of resting microglia and polarization toward different phenotypes through exosome delivery. In peripheral immune cells, miRNAs regulate the activation and extravasation of leukocytes by modulating signaling pathways. In addition, miRNAs regulate macrophage behavior in atherosclerosis, contributing to the etiology of IS. Furthermore, a considerable number of studies have shown that miRNAs serve as non-invasive, rapidly accessible, and low-cost biomarkers for diagnosing AIS with high sensitivity and specificity [20, 21, 23, 137]. The evidence also suggests that modifying specific miRNAs may represent a promising therapeutic approach for AIS.

However, there is a paucity of research on more comprehensive and precise molecular pathways targeted by miRNAs. It is crucial to identify specific miRNA targets. Since a single mRNA may bind multiple miRNAs, and one miRNA may target various mRNAs, potentially causing adverse effects. The CRISPR/Cas9 system is an effective tool for miRNA-related genome editing and regulation [138]. Moreover, contradictory outcomes across different studies, including variations in the detection of miRNA levels, highlight the need for further investigation. The credibility of the current study is limited by the sample size, and further validation in a larger risk-matched cohort is needed.

Moreover, miRNA therapy is also limited by low targeting efficiency, suboptimal stability, and insufficient cellular uptake. To translate biopharmaceutical research findings into clinical applications for IS, it is essential to design suitable dosage forms and determine optimal delivery methods. Intravenous administration is relatively safe but inefficient for CNS delivery due to the BBB. Direct administration of medication is effective but invasive and carries significant risks of adverse effects. Some studies suggest that intranasal administration may effectively cross the BBB with reduced injury in other CNS diseases [139]. Novel delivery strategies employing exosomes, liposomes, and lentiviruses may mitigate the risks associated with different routes, warranting further exploration [140, 141]. A recent study developed a tetrahedral framework nucleic acid-based delivery system, which allows miRNA-124 to enter cells efficiently. This delivery system has been proven to promote the transformation from M1 to M2, decreasing the infarct size [142]. Another group of researchers developed a novel DNA tetrahedral nanostructures-mediated HCR (DTN-HCR) platform for the precise detection of miR-25, for early detection of IS [143].

## AUTHORS’ CONTRIBUTIONS

The authors confirm their contributions to the paper as follows: Conceptualization - XS, validation - GJL, and drafting of manuscript - NHC, JXR. All authors reviewed the results and approved the final version of the manuscript.

## Figures and Tables

**Table 1:** Roles of miRNAs in brain-resident immune cells.

**Immune Cells**		**miRNAs**	**Mechanisms**	**Signaling Pathways or Targets**	**References**
Microglia	Activation of microglia	miR-424	Suppressing microglial activation and oxidative stress	Key activators of the G1/S phase transition Nrf2 pathway	[74, 75]
		miR-let-7c-5p	Decreasing microglial activation	caspase 3 pathway	[76]
		Inhibition of miR-155	Suppressing activated microglia	STAT-3 and JAK/STAT signaling cascade	[77, 78]
	Polarization of microglia	miR-155	Promoting both M1 and M2 polarization	TAB2; c-Maf	[79]
		miR-210	Promoting the inflammation of M1	SIRT1/ NF-κB pathway	[80, 81]
		miR-181c	Inhibiting pro-inflammatory cytokines	TLR4/ NF-κB pathway	[56, 82]
		miR-146a	Downregulating the expression of M1-related genes	IRAK1/ NF-κB pathway	[83]
		Upregulation of miR-145	Indicating the transformation from M0 to M2	-	[84, 85]
		Downregulation of miR-711	Indicating the transformation from M0 to M2	-	[84]
		Exosomes containing miR-124	Promoting polarization of microglia from M1 to M2	C/EBP-α and PU.1 USP14	[84, 86, 87]
		Exosomes containing miR-21-5p and miR-29b-3p	Promoting microglia polarization to M2 and inhibiting polarization to M1	mRNA expression of iNOS	[47]
		Exosomes containing miR-192-5p, miR-183, miR-378, miR-140-3p, miR-222	Promoting microglia polarization to M2	TNF and/or IL-1β	[88-91]
		miR-216-3p	Shifting microglia from M1 to M2 phenotype	TLR/ NF-κB signaling pathway	[92]

**Abbreviations**: Nrf2: nuclear factor erythroid 2-related factor 2; JAK-STAT: Janus kinase-signal transducer and activators of transcription; TAB2: transforming growth factor-β-activated kinase 1-binding protein 2; c-Maf: transcription factor MAF/proto-oncogene; SIRT1: Sirtuin1; NF-κB: Nuclear factor kappa B; TLR4: toll-like receptors 4; IRAK1: Recombinant Interleukin 1 Receptor Associated Kinase 1; C/EBP-α: CCAAT/enhancer binding protein-α; PU.1: protein Purine Rich Box-1; USP14: ubiquitin-specific protease 14; iNOS: inducible nitric oxide synthase;TNF: Tumor Necrosis Factor; IL-1β: Interleukin-1 beta.

**Table 2:** Roles of miRNAs in peripheral immune cells.

**Immune Cells**		**miRNAs**	**Mechanisms**	**Signaling Pathways or Targets**	**References**
Leukocytes	Activation of Leukocytes	miR-let-7i	Regulating leukocyte activation, recruitment, and proliferation	CD86 signaling in T helper cells, HMGB1 signaling and IL-8 signaling	[93]
		miR-122	Regulating immune activation	Expression of DAMPs	[94]
		miR-148	Altering cytokine production (IL-6, TNF-a, IL-12, CD70); T-cell proliferation; MHC II expression; DCs antigen presentation	Calcium/calmodulin-dependent protein kinase II	[94, 95]
		miR-16	Inhibiting macrophage activation	MAPK and NF-κB signaling; Programmed cell death 4	[25]
	Extravasation of Leukocytes	miR-148	Regulating the expression of the adhesion molecule LFA-1 and MMPs in leukocytes	Expression of DNA methyltransferase 1	[96]
		miR-19a	Modulating MMP-3; Facilitating angiogenesis	TLR2	[97]
		miR-let-7g	Attenuating the recruitment and migration of macrophages	Intracellular Ca2+-activated PKC-oxLDL-LOX-1 pathway	[25]
		miR-29b	Attenuating inflammatory response and augmenting BBB integrity	C1q and TNF-6	[98]
		Inhibition of miR-155	Suppressing monocyte adhesion and migration through the vascular endothelium	mRNA and protein expression of MCP-5 and CXCL3	[77]
Macrophages	-	miR-21	Increasing macrophage apoptosis and aggravating inflammation	p38/JNK/ERK signaling	[25]

**Abbreviations**: CD: Cluster of Differentiation; HMGB1: high mobility group box-1 protein; IL-8: Interleukin-8; DAMPs: danger-associated molecular patterns; TNF: tumor necrosis factor; MHC II: major histocompatibility complex II; DCs: dendritic cells; MAPK: mitogen-activated protein kinase; NF-κB: Nuclear factor kappa B; LFA-1: lymphocyte function-associated antigen 1; MMPs: matrix metalloproteinases; TLR2: Toll-like receptor 2; PKC: Protein Kinase C: oxLDL: oxidized low-density lipoprotein; LOX-1: lectin-like LDL receptor-1; C1q: Complement component 1q; MCP-5: monocyte chemotactic protein 5; CX3CL1: C-X3-C motif chemokine ligand 1; p38/JNK/ERK signaling: p38/ c-Jun N-terminal kinase/extracellular-signal-regulated protein kinase signaling.

**Table 3:** Utilizations of miRNAs as diagnostic biomarkers for AIS.

Biomarkers	miRNAs	Utilizations	Time Points	Sample	AUC Values	References
Diagnostic Biomarkers	The combination of three miRNAs (miR-125a-5p, miR-125b-5p, and miR-143-3p)	Increased in AIS patients compared with TIA patients and healthy controls	Increased within 24 hours after AIS, returning to normal levels within 48 hours after AIS	Plasma	0.90	[116]
	miRNA-221-3p	Decreased in AIS patients compared with healthy controls	Within 6 hours	Serum	0.810	[117]
	miRNA-382-5p	Decreased in AIS patients compared with healthy controls	Within 6 hours	Serum	0.748	[117]
	miR-124	Decreased in AIS patients compared with healthy controls	Decreased within 24 hours, increased within 72 hours	Serum	0.9527 (at 24 h), 0.9487 (at 48 h), 0.8668 (at 72 h)	[118]
	miR-30a	Decreased in AIS patients compared with healthy controls	Within 24 h, up to 24 weeks	Plasma	0.91	[119]
	miR-126	Decreased in AIS patients compared with healthy controls	Within 24 h, up to 24 weeks	Plasma	0.92	[119]
	let-7b	Downregulated in LAA but upregulated in other kinds of AIS patients	Within 24 h, up to 24 weeks	Plasma	0.93	[119]
	miR-125b	Increased in CARD patients compared with healthy controls	Within 24 h	Serum	0.906	[123]
	miR-125a	Increased in CARD patients compared with healthy controls	Within 24 h	Serum	0.866	[123]
	let-7b	Increased in CARD patients compared with healthy controls	Within 24 h	Serum	0.833	[123]
	let-7e	Increased in CARD patients compared with healthy controls	Within 24 h	Serum	0.923	[123]
	miR-family-17	Small vessel disease, but not in those with large artery, cardioembolic, or unclassified stroke	Within 48 h	Circulating extracellular vesicles	-	[122]
	miR-7-2-3p	Increased in LAA and LAC patients compared with healthy controls	Within 24 h	Serum	LAA: 0.874 LAC: 0.849	[121, 123]
	miR-1908	Decreased in LAA and LAC patients compared with healthy controls	Within 24 h	Serum	LAA: 0.811 LAC: 0.789	[123]

**Abbreviations:** AIS: acute phase of ischemic stroke; CARD: cardioembolic stroke; LAA: large-artery atherosclerotic stroke; LAC: lacunar stroke; TIA: transient ischemic attack area; AUC: under the curve.

**Table 4:** Potentials of miRNAs as therapeutic strategies for AIS.

**Therapeutic Strategies of miRNAs**	**Results in Preclinical Research**	**Models**	**References**
Mimics of miR-let-7c-5p	Reducing brain injury and neurological deficits	In MCAO mice	[76]
Activators of miR-29b	Reducing brain infarct volume and brain edema	In MCAO rats	[98]
Inhibitors of miR-377 and miR-200c	Reducing infarct volume and neurological impairment	In the MCAO rodent	[131, 132]
Inhibitors of miR-155	Supporting brain microvasculature, reducing brain tissue damage, and improving the animal's functional recovery	In the dMCAO mice	[77, 78]
Activators of miR-424	Reducing the cerebral infarction volume and brain edema	In MCAO mice	[75]
miR-124	Contributing to the restoration of brain cell function	In the stroke mouse model	[86]
Exosomes containing hsa-miR-124-3p and miR-30d-5p	Promoting microglia polarization to M2	In AIS patients and rat models	[135, 136]

**Abbreviations**: MCAO: middle cerebral artery occlusion; dMCAO: distal middle cerebral artery occlusion; AIS: acute phase of ischemic stroke.

## LIST OF ABBREVIATIONS

AIS: Acute Ischemic Stroke phase
AUC: Area Under the Curve
BBB: Blood-Brain Barrier
CD: Cluster of Differentiation
CNS: Central Nervous System
CT: Computed Tomography
DAMPs: Damage-Associated Molecular Patterns
DCs: Dendritic Cells
DWI: Diffusion-Weighted Imaging
FSRS: Framingham Stroke Risk Score
HT: Hemorrhagic Transformation
ICAS: Intracranial Atherosclerosis
IFN-γ: Interferon-Gamma
IGF-1: Insulin-like Growth Factor 1
IL: Interleukin
iNOS: Inducible Nitric Oxide Synthase
IS: Ischemic Stroke
JAK: Janus Kinase
LAA: Large-Artery Atherosclerotic stroke
LAC: Lacunar stroke
LPS: Lipopolysaccharide
MAPK: Mitogen-Activated Protein Kinase
MCAO: Middle Cerebral Artery Occlusion
MMPs: Matrix Metalloproteinases
mRNAs: messenger RNAs
miRNAs: microRNAs
NETs: Neutrophil Extracellular Traps
NF-κB: Nuclear Factor kappa B
NVU: Neurovascular Unit
OGD: Oxygen-glucose Deprivation
oxLDL: oxidized Low-density Lipoprotein
ROS: Reactive Oxygen Species
STAT: Signal Transducer and Activator of Transcription
TGF-β: Transforming Growth Factor-β
TIAs: Transient Ischemic Attacks
TLRs: Toll-like Receptors
TNF-α: Tumor Necrosis Factor-α
TOAST: Trial of Org 10172 in Acute Stroke Treatment
VCAM1: Vascular Cell Adhesion Molecule-1
VEGF: Vascular Endothelial Growth Factor
